# Supplementary material for: The interactome of intact mitochondria by cross-linking mass spectrometry provides evidence for coexisting respiratory supercomplexes
Source: Mol Cell Proteomics. 2017 Dec 8;17(2):216–32. doi: 10.1074/mcp.RA117.000470 (PMC5795388; doi:10.1074/mcp.RA117.000470)
Supplement: Supplemental Data [file supp_RA117.000470_133922_0_supp_23978_fzffwf.zip › spectra_annotation/mito_spectra_annotation/36-1-2-1-8-1.pdf]

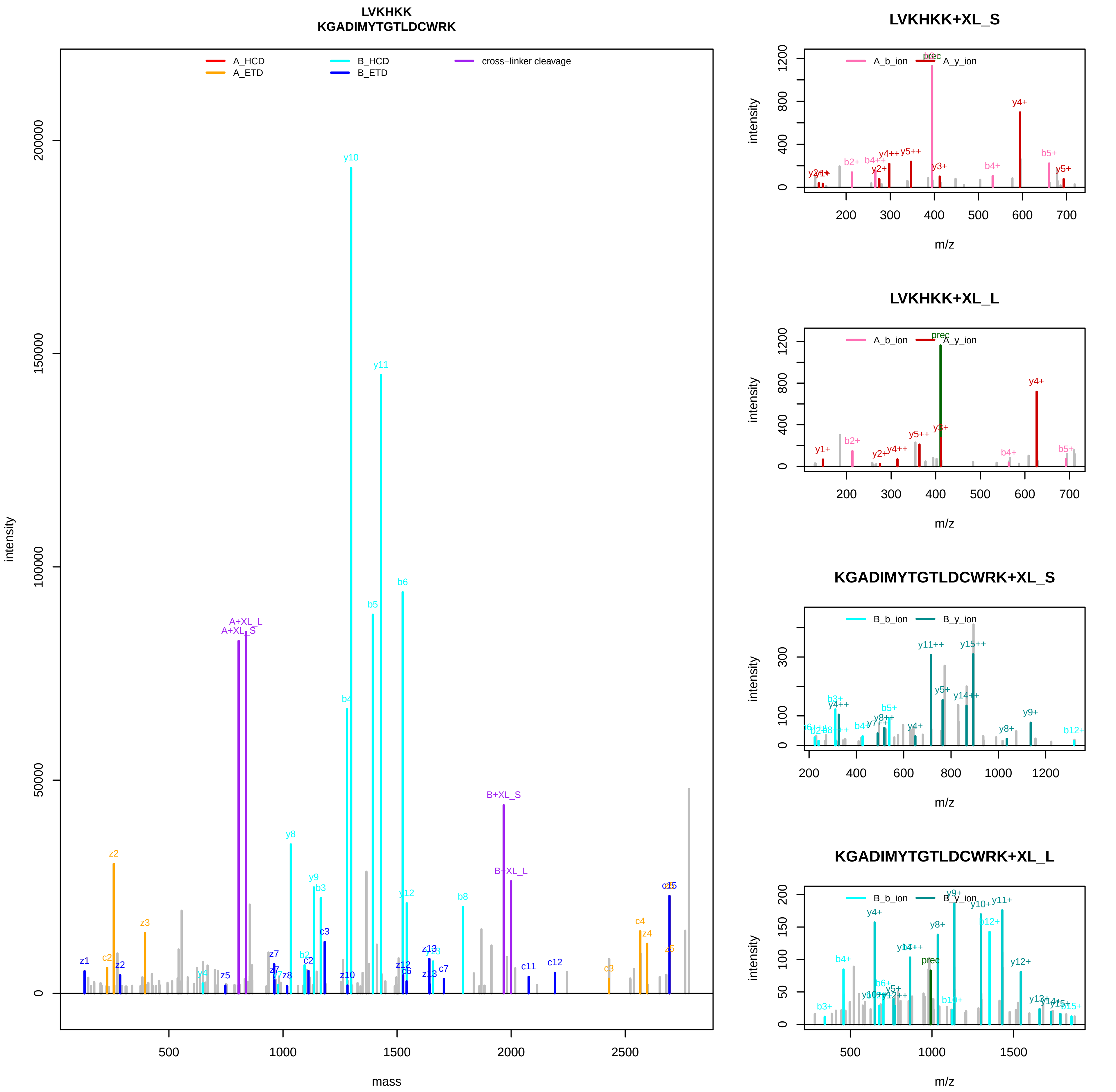
LVKHKK
KGADIMYTGTLDCWRK
A_HCD
A_ETD
B_HCD
B_ETD
cross−linker cleavage
intensity
mass
0
50000
100000
150000
200000
500
1000
1500
2000
2500
y10
y11
b6
b5
b4
A+XL_L
A+XL_S
B+XL_S
B+XL_L
y8
y9
b3
y12
b8
c15
z2
z3
c4
z4
LVKHKK+XL_S
LVKHKK+XL_L
KGADIMYTGTLDCWRK+XL_S
KGADIMYTGTLDCWRK+XL_L
A_b_ion
A_y_ion
B_b_ion
B_y_ion
prec
m/z
y4+
y5+
b5+
b4+
y3+
y5++
y4++
b2+
y2+
y1+
y11++
y15++
y14++
y9+
y8+
y10+
y12+
b12+
